# Diagnostic accuracy of evaluation of suspected syncope in the emergency department: usual practice vs. ESC guidelines

**DOI:** 10.1186/s12873-020-00344-9

**Published:** 2020-08-03

**Authors:** Veera K. van Wijnen, Reinold O. B. Gans, Wouter Wieling, Jan C. ter Maaten, Mark P. M. Harms

**Affiliations:** 1 Department of Internal and Emergency Medicine, University of Groningen, University Medical Center Groningen, PO BOX 30001, 9700 RB Groningen, the Netherlands; 2 Department of Internal Medicine, University of Amsterdam, Academic Medical Center, Amsterdam, the Netherlands

**Keywords:** Syncope, Emergency department, Diagnostic accuracy, History taking, Guidelines

## Abstract

**Background:**

Syncope is a frequent reason for referral to the emergency department. After excluding a potentially life-threatening condition, the second objective is to find the cause of syncope. The objective of this study was to assess the diagnostic accuracy of the treating physician in usual practice and to compare this to the diagnostic accuracy of a standardised evaluation, consisting of thorough history taking and physical examination by a research physician.

**Methods:**

This prospective cohort study included suspected (pre) syncope patients without an identified serious underlying condition who were assessed in the emergency department. Patients were initially seen by the initial treating physician and the usual evaluation was performed. A research physician, blinded to the findings of the initial treating physician, then performed a standardised evaluation according to the ESC syncope guidelines. Diagnostic accuracy (proportion of correct diagnoses) was determined by expert consensus after long-term follow-up.

**Results:**

One hundred and one suspected (pre) syncope patients were included (mean age 59 ± 20 years). The usual practice of the initial treating physicians did not in most cases follow ESC syncope guidelines, with orthostatic blood pressure measurements made in only 40% of the patients. Diagnostic accuracy by the initial treating physicians was 65% (95% CI 56–74%), while standardised evaluation resulted in a diagnostic accuracy of 80% (95% CI 71–87%; *p* = 0.009). No life-threatening causes were missed.

**Conclusions:**

Usual practice of the initial treating physician resulted in a diagnostic accuracy of 65%, while standardised practice, with an emphasis on thorough history taking, increased diagnostic accuracy to 80%. Results suggest that the availability of additional resources does not result in a higher diagnostic accuracy than standardised evaluation, and that history taking is the most important diagnostic test in suspected syncope patients. *Netherlands Trial Registration: NTR5651. Registered 29 January 2016,**https://www.trialregister.nl/trial/5532*

## Background

Suspected syncope is a frequent presenting symptom in the dynamic setting of the Emergency Department (ED) [1]. The first objective is to recognize a potentially life-threatening condition in the ED or ED observation unit [2]. After excluding a clear serious condition, the secondary objective is to find the cause of syncope. It is well known that physicians find it challenging to establish the cause of syncope in this large group of patients.

The European Society of Cardiology (ESC) developed the “Syncope Guideline on Diagnosis and Management” to reduce the risk of recurrences and the life-threatening consequences of syncope recurrences [3]. The initial evaluation consists of careful history-taking and physical examination, including an electrocardiogram and orthostatic blood pressure measurement. Despite the introduction of several syncope guidelines [1, 3–5], the current strategies and diagnostic yield of syncope evaluation varies widely between physicians, hospitals and countries [6]. To improve diagnostic yield (i.e., patients receiving a working diagnosis) several studies have applied standardised clinical evaluation, which has resulted in a working diagnosis of between 63 and 95% [7–9]. However, in daily practice, guideline adherence is not always synonymous with clinical practice [9]. Moreover, most studies did not provide data on the follow-up or prognosis of syncope patients presenting to the ED. Therefore, the diagnostic accuracy (proportion of correct diagnoses) of initial treating physicians in usual practice is unknown [9–11].

We performed a clinical audit to study the diagnostic accuracy of the usual evaluation of suspected syncope in the ED, after excluding a clear serious condition.

## Methods

### Setting

This prospective cohort study was conducted at a tertiary care hospital. The usual practice by the initial treating physician was compared to standardised practice by the research physician. Eligible patients had been referred to the ED from Monday to Friday during regular working hours due to a transient loss of consciousness that, on initial evaluation, was attributed to suspected severe presyncope or syncope. Syncope was defined as a transient loss of consciousness due to transient global cerebral hypoperfusion, characterised by rapid onset, short duration and spontaneous complete recovery [3]. Severe presyncope was defined as the feeling of almost losing consciousness, with similar prodromal symptoms as syncope [3, 12].

### Population

Patients were included between December 2015 and February 2017. Patients ≥18 years referred due to suspected syncope and severe presyncope were eligible for inclusion. Exclusion criteria were: a) hemodynamic instability, b) in need of immediate investigations/treatment (i.e., urgent work-up by attending staff, whereby the research process would delay diagnostic/therapeutic care), c) psychologically, physically (due to a serious illness) or cognitively unfit, d) unable to participate in the follow-up study, e) unwilling or unable to give informed consent, f) transient loss of consciousness not fitting the definition of suspected syncope, or g) a life expectancy of less than 1 year. The study was approved by the Medical Ethical Committee and all patients provided informed consent.

### Study flow

#### Practice as usual by initial treating physicians

**Fig. 1:**
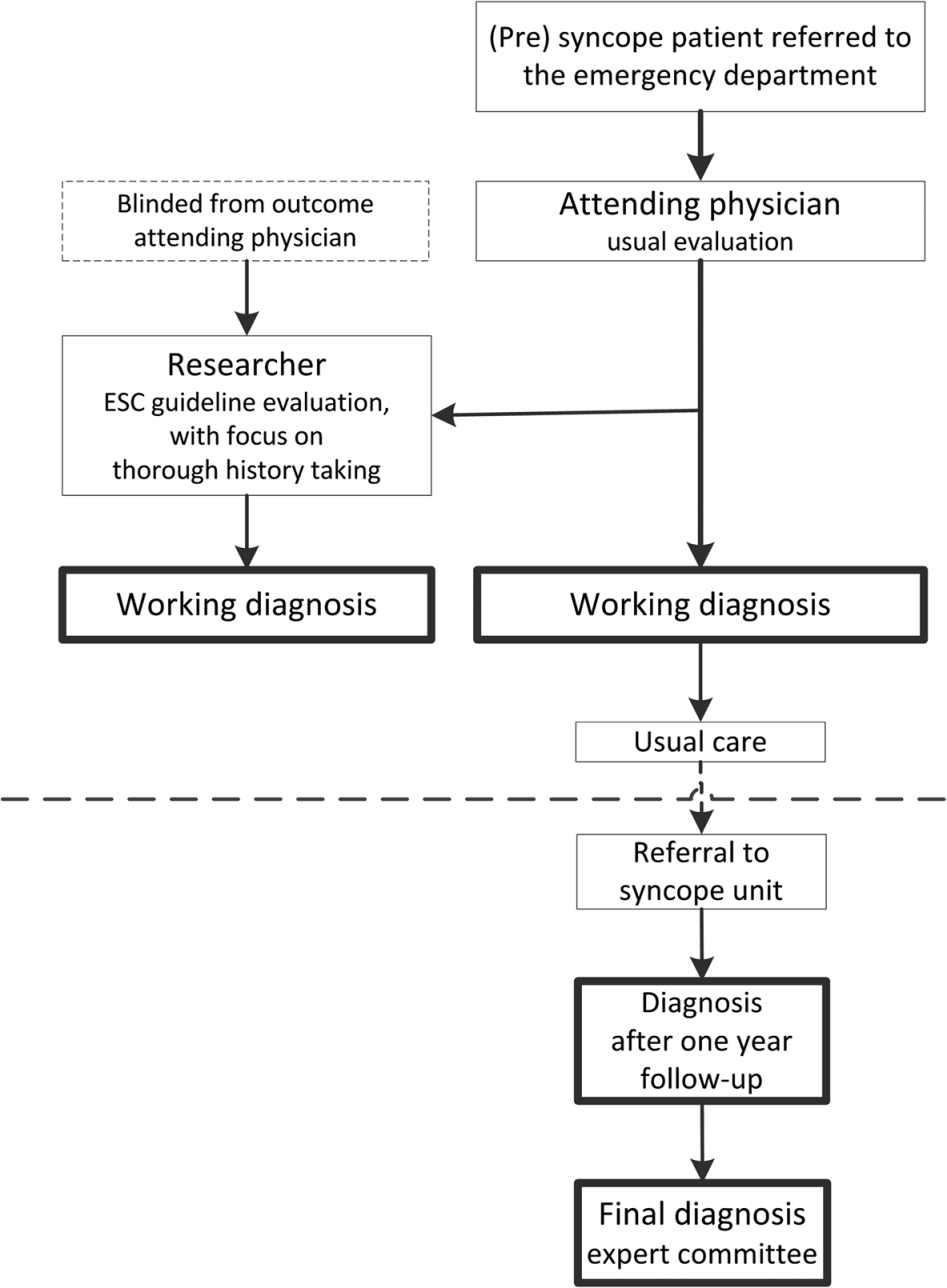
Study flow diagram

Patients who were referred to the ED and were seen by the Emergency Medicine, Internal Medicine and Cardiology specialists were screened for eligibility to participate in the study. First, patients were assessed by the initial treating physicians, who were usually residents under the supervision of a specialist. The evaluation of a patient with suspected syncope forms part of the resident training program, whereby the ESC guidelines form the standard of care [3]. In addition to the existing resident training program, no specific guidelines were given to the initial treating physicians and therefore the evaluation was a reflection of usual practice (Fig. 1). The working diagnosis given by the initial treating physician was mined from the ED discharge letter, signed by the resident and supervisor (the initial diagnosis included in the letter’s conclusion).

#### Standardised evaluation by the research physician

After the usual evaluation, the initial treating physician alerted the full-time research physician, who approached the patient to obtain informed consent. The initial treating physicians were explicitly instructed not to reveal their working diagnosis to the research physician. Moreover, the initial treating physicians were unaware of the specific aim of the study. After obtaining informed consent, an extensive history [13] and physical examination, including the analysis of the electrocardiogram and orthostatic BP measurement was performed by the research physician [3]. The research physician, a physician with an in-depth knowledge of orthostatic blood pressure regulation, was trained by a syncope expert to take a standardised in-depth history following the ESC syncope guideline [3, 13, 14].

### Follow-up

The initial management of (pre) syncope patients in the ED was independent of this study and determined by the initial treating physicians. Nevertheless, the initial treating physicians were aware that all patients received a referral to the syncope unit. During the follow-up visit(s) at the syncope unit, additional investigations were performed if this was appropriate (outside the scope of this article). If patients did not present for their appointment, they were contacted by phone or via their General Practitioner at the end of the year to obtain further information regarding recurrences and/or additional investigations. The diagnosis was determined at the syncope unit after one-year follow-up. We also classified the clinical certainty of the diagnosis that was made, according to Van Dijk et al. [7], into a *certain* (100%), *highly likely* (80–100%), *likely* (60–80%) or *uncertain* (< 60%) diagnosis. A *certain* diagnosis met the criteria as described by van Dijk et al. [7].

### Reference standard

To determine diagnostic accuracy (proportion of diagnoses in the correct diagnostic category), a reference standard was required. As no independent reference standard for syncope exists [13], all information obtained during long-term follow-up was used to test the reliability of the diagnosis through assessment by an independent expert committee [7, 14]. To obtain the final diagnosis, the following procedure was used: Patients who received the same diagnosis by the initial treating physician, the research physician and after one-year of follow-up (defined as *certain* or *highly* likely), were classified as having received the correct diagnosis. All other cases were reviewed by the expert committee.

### Expert committee

The expert committee consisted of three independent syncope specialists (a cardiologist, neurologist, and an internist) with many years of experience in the field [15–17]. The experts were not members of the study group. The expert committee reviewed all available information summarised per patient and decided upon a final diagnosis. When all experts agreed on a diagnosis or two members agreed on a diagnosis and the third member made no diagnosis, this diagnosis was taken as the final diagnosis. Finally, a face-to-face consensus meeting was held to discuss the cases in which no agreement had been reached. If an agreement was then reached, this was designated the final diagnosis [7].

### Outcomes

#### Diagnostic accuracy

Diagnostic accuracy was defined as the proportion of correct diagnosis after the initial evaluation in the correct diagnostic category (reflex syncope, cardiac syncope, initial orthostatic hypotension, orthostatic hypotension, psychogenic pseudosyncope, or non-syncope (e.g., hyperventilation, hypoglycemia, epilepsy) [7].

#### Adverse events

Adverse events were defined as death, life-threatening arrhythmia, acute myocardial infarction, severe structural heart disease, aortic dissection, pulmonary embolus, cerebrovascular accident, subarachnoid hemorrhage, significant hemorrhage, another severe condition or procedural interventions, which occurred within 30 days after referral to the ED [18].

### Statistical analysis

IBM SPSS Statistics 23 was used for analyses. Continuous data were expressed as mean (SD) or median (interquartile range) where appropriate, and categorical data as percentages. Between-group differences in continuous data were analysed using Student’s t-test or nonparametric tests where appropriate. Diagnostic accuracy was expressed in proportions and the method by Wilson was used to calculate 95% confidence intervals. Statistical significance was set at *p* < 0.05.

## Results

### Patient characteristics

Two hundred and 18 suspected syncope patients were screened for inclusion. Of these patients, 117 (53.7%) were ineligible to participate due to refusal to participate (*n* = 38) or because of the presence of an exclusion criterion (*n* = 79). The reasons for exclusion were hemodynamic instability (*n* = 5), in need of immediate investigations/treatment (*n* = 17), psychologically, physically or cognitively unfit (*n* = 31), unable to participate in the follow-up study (*n* = 8), transient loss of consciousness not fitting the definition of suspected syncope (*n* = 11), a life expectancy of less than 1 year (n = 3), or the presence of a language barrier (*n* = 4). The differences observed between the included and excluded patient groups were found in the suspected diagnosis categories, admission rate and all-cause mortality (Table 1).

**Table 1:** Group characteristics of suspected (pre) syncope patients in the Emergency Department

	Included *N* = 101	Excluded *N* = 117	***P*** value
**Demographics**			
Male, *n* (%)	66 (65.3)	62 (53.0)	0.065
Age, mean ± SD	59 ± 20	61 ± 21	0.280
**Specialists in the ED**, *n* (%)			0.177
•Emergency Medicine	62 (61.4)	58 (49.6)	
•Internal Medicine	14 (13.9)	27 (23.1)	
•Cardiology	25 (24.8)	32 (27.4)	
**Diagnosis by initial treating physician,***n* (%)			< 0.001*
•Vasovagal reflex syncope	54 (53.5)	36 (30.8)	
•Situational reflex syncope	3 (3.0)	2 (1.7)	
•Carotid sinus hypersensitivity	–	–	
•Cardiac syncope	10 (9.9)	27 (23.1)	
•Initial orthostatic hypotension	–	–	
•Orthostatic hypotension	14 (13.9)	14 (12.0)	
•Psychogenic pseudosyncope	–	–	
•Other cause, non-syncope	7 (6.9)	10 (8.5)	
•Unknown	13 (12.9)	28 (23.9)	
**Admission**, *n* (%)	16 (15.8)	53 (45.3)	< 0.001*
**All-cause mortality,***n* (%)			
•30 days	–	3 (2.6)	–
•1 year	5 (5.0)	18 (15.4)	0.005*

*ED* Emergency Department. *Statistically significant at *p* < 0.05.

The included 101 patients had a mean age of 59 ± 20 years and 66 (65.3%) were male. Twenty-nine (28.7%) patients were referred due to severe presyncope and 72 (71.3%) patients due to syncope. Fifty-four (53.5%) patients had a history of cardiovascular disease and 63 (62.4%) a history of syncope (Table 2).

**Table 2:** Additional patient characteristics of included (pre)syncope patients

**Arrival by ambulance**, n (%)	71 (71.0)
**Cardiovascular history**, n (%)	54 (53.5)
• Hypertension	40 (39.6)
• Myocardial infarction	14 (13.9)
• Heart failure	4 (4.0)
• Rhythm disorders (AF, VT)	15 (14.9)
• Pacemaker/ICD	5 (5.0)
• Peripheral vascular disease	9 (8.9)
• Thrombosis	8 (7.9)
Parkinson’s disease	3 (3.0)
Diabetes mellitus	12 (11.9)
History of syncope	63 (62.4)
**Antihypertensive medication**, n (%)	46 (45.5)
• B-blocker	25 (24.8)
• ACE inhibitor or angiotensin II blocker	31 (30.7)
• Calcium antagonist	9 (8.9)
• Diuretics	21 (20.8)
• ≥2 antihypertensive drugs	29 (28.7)
Antidepressants	8 (7.9)
Polypharmacy (≥5)	40 (39.6)
**ECG performed**	101 (100.0)
**Orthostatic BP test performed by initial treating physicians**	40 (39.6)
**Additional tests performed in the ED**, n (%)	
• Laboratory tests	97 (96.0)
• Chest x-ray	18 (17.8)
• Computed tomography (head or chest)	14 (13.8)
**Consultation of another specialist in the ED**, n (%)	33 (32.7)
• One specialist	27 (26.7)
• Two specialists	6 (5.9)
**Department of admission**, n (%)	
• Cardiology	5 (31.2)
• Internal Medicine	10 (62.5)
• Neurology	1 (6.3)
**Median number of days hospitalised** (range)	3.13 (1-13)

*AF* atrial fibrillation, *BP* blood pressure, *ED* Emergency Department, *ICD* implantable cardioverter-defibrillator, *VT* ventricular tachycardia

### Usual practice by the initial treating physicians

The initial evaluation by the initial treating physicians consisted of a history and physical examination, including an electrocardiogram in 100% of the patients (Table 2). Orthostatic blood pressure measurement was performed in 40/101 (39.6%) patients. Furthermore, laboratory tests were performed in 97 (96.0%) patients, chest x-ray in 18 (17.8%) and computed tomography (head or chest) in 14 (13.8%). Another specialty was consulted for 33/101 patients. No adverse events occurred within 30 days.

### Diagnostic accuracy of the diagnosis at presentation

#### Expert committee

Forty-two (41.6%) patients were assessed by the expert committee. In 19/42 patients, the experts agreed during the paper round. The other 23/42 patients were discussed during a face-to-face meeting. Finally, seven patients did not receive a diagnosis.

#### Diagnostic accuracy of the initial treating physicians and research physician

**Fig. 2:**
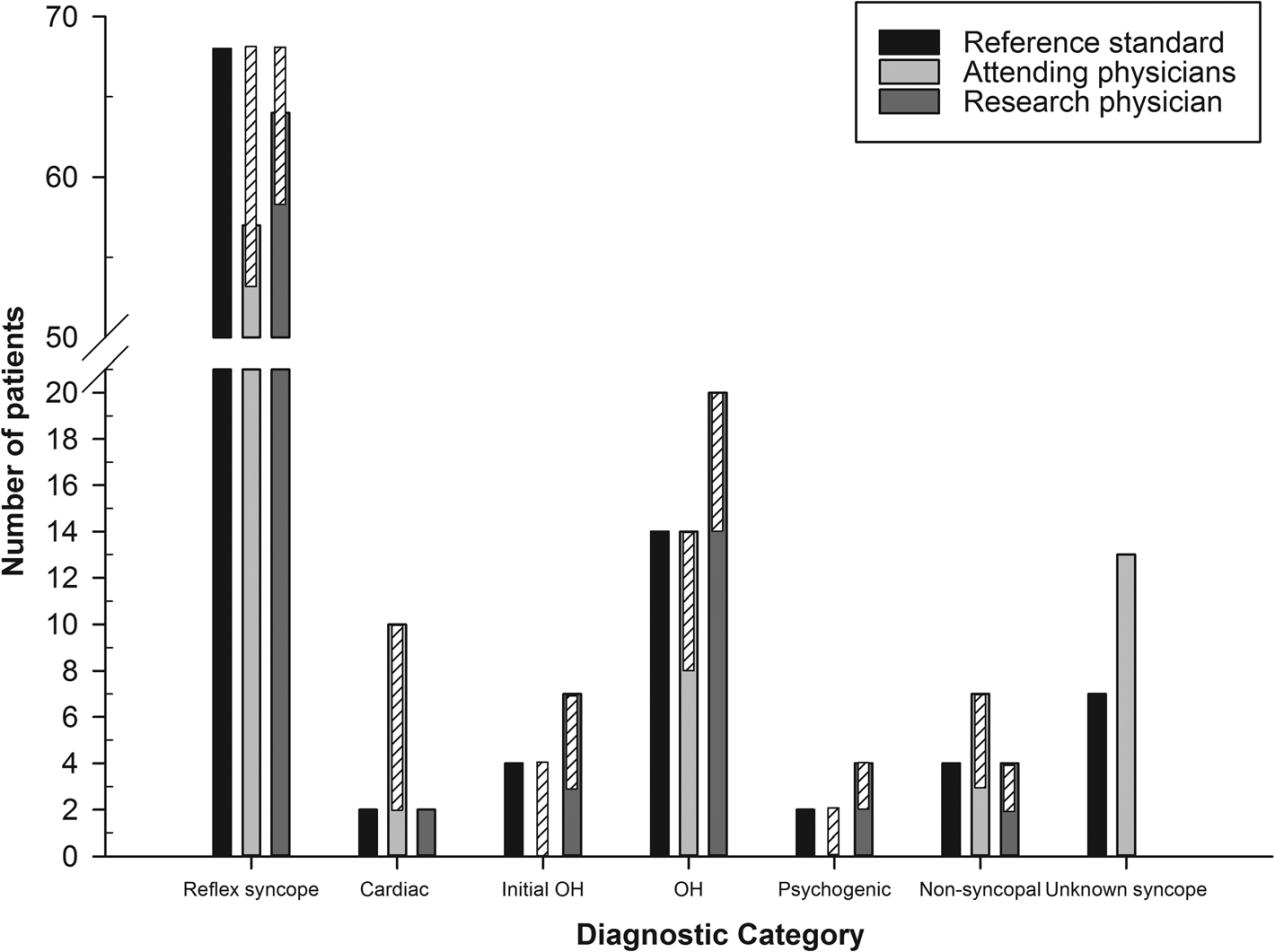
Diagnostic accuracy of the initial treating physicians and research physician against the reference standard. The bar with slashes represents the total misdiagnoses by the initial treating physicians and research physician. Vasovagal and situational syncope were grouped together under reflex syncope

The diagnostic accuracy of the initial treating physicians was 65% (95% CI: 56–74%) and of the research physician 80% (95% CI: 71–87%; *p* = 0.009; Table 3, Fig. 2). The diagnosis of the initial treating physicians corresponded to the diagnosis of the research physician in 59/66 cases (89.4%).

**Table 3:** Diagnostic accuracy of initial treating physicians and research physician

Reference standard										
		vasovagal reflexsyncope	situational reflex syncope	cardiac syncope	Initial OH	OH	psychogenic pseudosyncope	non syncopal attack	Unknown	Total initial treating physicians
**Initial treating physicians**	vasovagal reflex syncope	**47**	**3**	–	–	2	1	1	–	**54** (53.5%)
	situational reflex syncope	**1**	**2**	–	–	–	–	–	–	**3** (3.0%)
	cardiac syncope	3	–	**2**	2	2	–	–	1	**10** (9.9%)
	Initial OH	–	–	–	–	–	–	–	–	**–**
	OH	3	–	–	2	**8**	–	–	1	**14** (13.9%)
	psychogenic pseudosyncope	–	–	–	–	–	–	–	–	**–**
	non syncopal attack	3	–	–	–	–	–	3*	1	7* (6.9%)
	Unknown	6	–	–	–	2	1	–	**4**	**13** (12.9%)
**Total Reference standard**		**63** (63.4%)	**5** (5.0%)	**2** (2.0%)	**4** (3.9%)	**14** (13.9%)	**2** (2.0%)	4* (3.9%)	**7** (6.9%)	**101**
**Reference standard**										
		vasovagal reflex syncope	situational reflex syncope	cardiac syncope	Initial OH	OH	psychogenic pseudosyncope	non syncopal attack	unknown	**Total Researcher**
**Researcher**	vasovagal reflex syncope	**50**	–	–	–	–	–	1	4	**55** (54.5%)
	situational reflex syncope	**3**	**5**	–	1	–	–	–	–	**9** (8.9%)
	cardiac syncope	–	–	**2**	–	–	–	–	–	**2** (2.0%)
	Initial OH	2	–	–	**3**	–	–	–	2	**7** (6.9%)
	OH	5	–	–	–	**14**	–	1	–	**20** (19.8%)
	psychogenic pseud osyncope	2	–	–	–	–	**2**	–	–	**4** (3.9%)
	non syncopal attack	1	–	–	–	–	–	**2**	1	**4** (3.9%)
	Unknown	–	–	–	–	–	–	–	–	–
**Total Reference standard**		**63** (63.4%)	**5** (5.0%)	**2** (2.0%)	**4** (3.9%)	**14** (13.9%)	**2** (2.0%)	4* (3.9%)	**7** (6.9%)	**101**

Diagnostic accuracy is defined as the proportion of patients with a diagnosis after initial evaluation in the correct diagnostic category (using expert consensus after long-term follow-up). In both tables, the reference standard represents that correct diagnostic category. The upper table compares the working diagnosis made by the initial treating physicians to the reference standard. The lower table compares the working diagnosis made by the researcher with the reference standard. *Indicates one patient diagnosed with epilepsy. OH= orthostatic hypotension

#### Diagnostic accuracy of the diagnostic categories

A diagnosis of reflex syncope, combining vasovagal and situational syncope, was the most common diagnosis (Table 3, Fig. 2). Diagnostic accuracy for the diagnosis of reflex syncope was 78% (95% CI: 67–86%) for the initial treating physicians and 85% (95% CI: 75–92%) for the research physician. No cardiac syncope was missed. However, eight patients received a working diagnosis of cardiac syncope from the initial treating physicians, while the expert committee categorised these patients differently. Initial orthostatic hypotension was the cause of (pre) syncope for four patients; however, no diagnosis of initial orthostatic hypotension was made by the initial treating physicians. Orthostatic hypotension was the second most common cause of syncope (14%); the diagnostic accuracy of the initial treating physician and the research physician were 57% (95% CI: 33–79%) and 100% (95% CI 78–100%), respectively. However, the research physician misclassified six patients with presumed orthostatic hypotension. Finally, two patients were classified by the experts as having psychogenic pseudosyncope. The initial treating physicians did not classify any patients with this diagnosis.

#### Diagnostic accuracy after one-year follow-up

In addition to the diagnostic accuracy of the initial evaluation in the ED, the accuracy of the diagnosis made after 1 year of follow-up was also assessed. Diagnostic accuracy after one-year follow-up was 89% (95% CI: 81–94%) (*p* < 0.001 and *p* = 0.012 comparing the initial treating physicians and research physician in the ED, respectively). Of the patients who received a *certain* diagnosis (*n* = 34), one patient was judged differently by the expert committee. In the group with *highly likely* diagnoses (*n* = 48), one diagnosis was different according to the expert committee. In the category of *likely* diagnosis, eight patients received another diagnosis by the expert committee.

## Discussion

In this clinical audit, the diagnostic accuracy of the usual evaluation of suspected syncope in the ED was assessed, after excluding a clear serious condition. The usual method of syncope evaluation by initial treating physicians in the ED resulted in a diagnostic accuracy of 65%. A diagnostic accuracy of 80% was achieved after a standardised evaluation by the research physician, with the emphasis on thorough history-taking. The availability of additional resources (tests and/or consultations) did not result in a higher accuracy than the standardised assessments.

### Diagnostic accuracy of the usual method of practice by initial treating physicians

Although the ESC syncope guideline is the standard taught in residents’ training programs, the usual method of evaluation by the initial treating physicians did not, in most cases, proceed according to the ESC guidelines [3]. Orthostatic blood pressure measurement was performed in only 40% of patients, and in addition many additional tests (96%) and consultations (33%) were performed. This is the first study to determine the diagnostic accuracy of the usual practice of initial treating physicians using expert consensus as the reference standard after long-term follow-up. Previous studies have focused on (increasing) diagnostic yield [8, 9, 19]; whether the working diagnosis was correct or incorrect was usually neglected [7]. Van Dijk and colleagues were the first to assess the diagnostic accuracy of the initial treating physicians in a tertiary ED, applying a standardised-care pathway in the evaluation of 112 suspected syncope patients [7]. They found that the initial evaluation was performed according to ESC guidelines, resulted in an overall diagnostic accuracy of 70% [7]. This percentage is slightly higher than the non-standardised evaluation observed in our ED. However, the populations of these two studies cannot directly be compared, as the mean age of patients in the study by Van Dijk was considerably lower (45 ± 18 vs. 59 ± 20 years) and subjects were less likely to have cardiovascular comorbidities. However, the results of these two studies suggest that standardised and non-standardised care (with use of additional resources) by treating physicians in the ED results in a diagnostic accuracy between 65 and 70%.

### Diagnostic accuracy after standardised evaluation

The findings of this study suggest that a standardised evaluation, with the emphasis on thorough history-taking, will further improve diagnostic accuracy in the evaluation of syncope patients in the ED. The importance of history taking in syncope patients has previously been addressed; however, these studies have usually referred to history taking by syncope experts in syncope units [13, 14, 20]. To improve history taking, physicians need to be able to link clinical clues from the medical history to physiology [21]. The key to successful syncope history-taking is to allow sufficient time to carefully listen to the patient with undivided attention [13]. Moreover, it is important to realise that reflex syncope and orthostatic hypotension are the most common causes of syncope, and therefore physicians caring for patients with suspected syncope should have an understanding of circulatory physiology and pathophysiology [22]. Following on from this, orthostatic vital signs are indicated in all suspected syncope patients. We argue that to obtain the diagnosis of syncope, a thorough history and physical examination by a capable physician is often all that is necessary to reach a diagnosis and begin therapy [20].

### Clinical relevance of improved diagnostic accuracy

An accurate diagnosis is important in order to explain the mechanism of the episode to the worried patient and to determine the correct management and follow-up [14]. It can be argued that an evaluation within the dynamic setting of the ED is focused on risk stratification, and it is true that life-threatening causes must first be excluded. However, patients need more reassurance than “it’s not epilepsy or your heart” [15]. Furthermore, distinguishing between the diagnostic categories is important due to varying associated risks. Cardiac syncope carries the highest risk, and admission and treatment of the underlying cause here is crucial [3, 23]. In addition, orthostatic hypotension is also related to an increased risk of serious outcomes within the first 30 days after ED evaluation, and is generally associated with an increased risk of cardiovascular morbidity and mortality [24, 25]. Patients with vasovagal reflex syncope are not at an increased risk of death; however, unexplained syncope and recurrent reflex syncope are associated with excessive diagnostic testing and decreased quality of life [24, 26].

In addition, initial orthostatic hypotension and psychogenic pseudosyncope are recognised causes of suspected syncope in the ED, but are under recognised by most attending physicians. In young subjects with initial orthostatic hypotension, simple and effective advice can be given to prevent new episodes (counter pressure manoeuvers) [27]. In elderly subjects, initial orthostatic hypotension with a delayed recovery of BP has been recognised as a cause of (pre) syncope shortly after standing up [28]. Finally, psychogenic pseudosyncope is important to diagnose as patients then require specialty consultation. A combination of specific features from the history usually reveals the diagnosis to the attending physician who is alert to it.

### Final diagnosis after one year of follow-up

The reference standard used in this study, namely expert consensus after one-year of follow-up, is the best reference standard available, but is imperfect [3, 14]. Long-term follow-up has the advantage of providing more information regarding additional tests, recurrences and morbidity, but simultaneously it can make assessment of the ‘first’ episode harder for the expert committee. Moreover, the expert committee may be less likely to categorise borderline cases as cardiac, as they do not have the responsibility of patient care.

In previous studies without expert consensus, very strict criteria were used to define a ‘certain’ diagnosis, leading to lower number of patients with a diagnosis [4]. Therefore, Van Dijk et al. advocated that accepting more uncertainty would increase diagnostic yield and diagnostic accuracy, and extensive testing can be avoided [7].

The results of this study characterise the challenges that arise in diagnosing patients with syncope and determining diagnostic accuracy. The results imply that physicians dealing with suspected (pre) syncope patients on a regular basis should gather sufficient knowledge of historical clues and physical findings to recognise major causes of transient loss of consciousness (including mimics) and syndromes of orthostatic intolerance [22, 29]. There should be an emphasis on thorough history taking when discussing ways to improve syncope practice and knowledge.

### Limitations

Several limitations affected generalisability and replicability. The study population was a selected population from the ED of a tertiary teaching hospital and patients with an identified serious condition were excluded. This is the likely explanation that there were no adverse events within 30 days of follow-up. However, the age and cardiovascular comorbidity of this population was comparable to a large Italian multi-center study of syncope patients [9]. Furthermore, as this study compared the initial evaluation of many treating physicians, it is a true representation of daily practice in an ED in the Netherlands. However, this makes replication of the study difficult. The diagnostic accuracy of the diagnosis made after 1 year of follow-up at the syncope unit was high, but not 100%. This indicates that, despite follow-up at a syncope unit, a group of patients will remain difficult to classify. Lastly it is important to address the possibility of bias, the so-called Hawthorne effect. Even though the treating physicians were not aware of the study aim, it is possible that they modified their assessment because of their awareness of being observed, and therefore performing better than generally.

## Conclusion

The usual evaluation of suspected syncope in the ED by the initial treating physician resulted in a diagnostic accuracy of 65%, while standardised assessment according to the ESC guideline with an emphasis on thorough history-taking increased diagnostic accuracy to 80%. These results suggest that the usual practice has a lower diagnostic accuracy, and that applying the ESC guidelines could result in less testing being required to obtain the correct diagnosis.

## Abbreviations

ED: Emergency Department
ESC: European Society of Cardiology
OH: Orthostatic Hypotension
VVS: Vasovagal Syncope
